# A study on dietary diversification intervention for elderly patients with chronic heart failure who can eat by mouth

**DOI:** 10.3389/fnut.2026.1714510

**Published:** 2026-05-13

**Authors:** Wenlin Zhou, Longya Si, Yumei Deng, Jing Li, Changxiu Li

**Affiliations:** 1 Department of Nursing, The Affiliated Hospital of Zunyi Medical University, Zunyi, Guizhou, China; 2 School of Nursing, Zunyi Medical University, Zunyi, Guizhou, China; 3 Department of Nursing, The First People’s Hospital of Yunnan Province, Kunming, Yunnan, China

**Keywords:** chronic heart failure, dietary diversity, elderly, intervention program, oral intake

## Abstract

**Introduction:**

Conventional CHF nutrition counseling is often one-size-fits-all. We developed and tested a DDS-guided, tiered dietary diversification program for elderly CHF patients able to eat orally.

**Method:**

A quasi-experimental study with a non-randomized, non-concurrent control design was used. Eighty-eight elderly oral-feeding CHF patients were selected from the Cardiovascular Department of a tertiary general hospital in Zunyi City (July–October 2022): the control group (July–August, *n* = 44) and the intervention group (September–October, *n* = 44). The control group received conventional dietary guidance, while the intervention group underwent personalized intervention based on the Dietary Diversity Score (DDS9) and nutritional status, with 2-month post-discharge follow-up. The Dietary Diversity Score (DDS9) and nutritional indicators (Heart Failure-Specific Mini Nutritional Assessment [MNA-HF], Body Mass Index BMI) were compared between the groups at baseline, 1 and 2 months post-intervention.

**Results:**

There were no statistically significant differences in baseline data between groups (*p* > 0.05). After 1 month, the intervention group showed significantly higher scores in Total DDS and specific food groups (fish, fruits, etc.) compared to the control group (*p* < 0.05), with a large effect size for Total DDS. After 2 months, significant improvements were maintained for Total DDS (large effect size) and MNA-HF (medium effect size) (*p* < 0.05). The intervention group exhibited a significantly greater upward trend in dietary diversity over time (*p* < 0.001, large effect size). Although absolute BMI trends did not differ significantly, the increase in BMI at 2 months was significantly greater in the intervention group (medium effect size).

**Discussion:**

A DDS-guided, tiered intervention improved dietary diversity and nutritional status over 2 months in elderly CHF patients, demonstrating practicality and scalability. Limitations include the non-randomized, single-center design and short follow-up; longer multicenter trials are needed to confirm durability and clinical impact.

## 1 Introduction

Heart failure (HF) is a complex clinical syndrome characterized by structural and/or functional abnormalities of the heart caused by various etiologies, leading to impaired ventricular systolic and/or diastolic function. This results in symptoms primarily manifested as dyspnea, fatigue, and fluid retention (pulmonary congestion, systemic congestion, peripheral edema, etc.) (1, 2). Chronic heart failure (CHF) refers to a persistent state of HF, encompassing stable, worsening, or decompensated phases (3). In developed countries, the prevalence of heart failure ranges from 1.0 to 2.0% (4, 5). In China, the prevalence stands at 1.1%, and with an aging population, this rate continues to rise, exceeding 10% among those aged ≥70 years (6, 7). However, rehospitalization and mortality rates remain high among elderly CHF patients: 83% experience at least one hospitalization within a year, 43% require four or more hospitalizations, and 1-year and 5-year mortality rates after diagnosis are 20 and 53%, respectively (4, 8). According to the 2021 China Cardiovascular Health and Disease Report (9). Hospitalization costs for CHF patients reached 13.064 billion yuan in 2019, with elderly patients accounting for 85% of these expenses, a proportion that increases with age. Total HF costs are projected to rise to $69.8 billion by 2030 (8). The high prevalence, readmission rates, and mortality among elderly CHF patients impose a substantial healthcare burden, making them a critical focus in global cardiovascular disease prevention and management.

Research shows that (10), the risk of malnutrition is higher than 50% for older CHF patients. It’s common to ignore the nutritional status of elderly CHF patients who consume oral food, and malnutrition has several detrimental effects. Malnutrition causes an overabundance of cardiac autophagy, which has negative consequences on cells. By eliminating cytoplasmic elements such as proteins and senescent organelles, normal cardiac autophagy prolongs the life of cardiomyocytes while recycling useful components for the creation of new proteins. But in cases of starvation, excessive cardiac autophagy may eliminate molecules and organelles necessary for the survival of cardiomyocytes, which could result in organ failure and patient death (11). Further aggravation of condition (12) may result from malnutrition’s numerous processes of exacerbating cardiac pathological remodeling. Macro-level evidence suggests that malnutrition in older patients with congestive heart failure is directly linked to negative outcomes, such as higher rates of adverse cardiovascular events and readmissions (13), longer hospital stays and higher hospitalization expenses (14), lower quality of life (15), and an increased risk of death (16, 17). Nutritional treatment is a crucial intervention for older CHF patients to increase exercise tolerance, alleviate symptoms, improve quality of life, lower readmission rates, and lower mortality, according to recent expert consensus statements and guidelines (3, 18).

Low-salt dietary management is currently the mainstay of traditional nutritional interventions for elderly CHF patients receiving oral feeding (19, 20). However, more research has shown that strict low-salt diets activate the renin-angiotensin-aldosterone system, increasing the risk of mortality, rehospitalization, and symptom deterioration (21). Low-salt diets may also make food less palatable for elderly CHF patients, reducing food intake and increasing the risk of malnutrition while lowering quality of life (22). And that individualized nutritional support for HF patients, involving dietitian participation, can improve nutritional status, lower the risk of all-cause mortality, increase functional status, and improve quality of life (23). Nutritional counseling and dietitians’ full-process involvement in individualized nutritional support are the main forms of personalized nutritional interventions in certain developed nations. However, because medical resources are limited in China, professional dietitians are frequently not fully involved in the process of developing individualized nutritional therapies for elderly patients with congestive heart failure. While 95.05% of patients with chronic heart failure were advised by their doctors to follow a low-salt diet, only 9.83% of them strictly followed the recommendations, according to a survey study (24). Patients are less likely to adhere to traditional nutrition education since it is often ambiguous and abstract. This is especially troublesome for older CHF patients who have less education because they could find it difficult to understand dietary guidelines (10, 25). As a result, their attitudes and actions about nutrition continue to be below ideal. An analysis of a qualitative study, for example (26) found that HF patients’ poor adherence to low-salt diets was caused by their ignorance of the benefits of low-salt diets, food choices, and preparation techniques. The eating habits of CHF patients are still worrisome, though (27). Whether at home or in the hospital, patients and their families must prioritize nutritional advice.

The survey found that older CHF patients in western regions only followed dietary management guidelines, which only included avoiding high-sodium foods or reducing salt intake, avoiding high-fat, high-cholesterol foods, and avoiding overeating, at a rate of 66.67%. This was lower than the rates in eastern, southern, northern, and central China (28). This discrepancy may result from both the influence of specific distinct lifestyle practices and ethnic customs among minority patients as well as worse medical and economic conditions in western regions (29). As a western province, Guizhou has to deal with issues such doctors’ lack of nutritional expertise and older CHF patients’ lower educational attainment, lower socioeconomic standing, and generally worse comprehension skills. In this regard, elderly CHF patients in Guizhou who are still able to eat orally could have their nutritional status and prognosis considerably improved by offering clear, concise, and comprehensive dietary suggestions.

In order to deliver vital nutrients and promote optimal dietary quality and health outcomes, dietary diversity (DD) refers to expanding the range of foods both within and between food groups (30). DD is acknowledged as a straightforward, efficient, and reasonably priced nutritional intervention (31, 32). And is an essential part of a healthy diet. DD helps older persons maintain and increase their nutritional status while reducing their risk of malnutrition (33). 6,587 older persons participated in a major cross-sectional study (34) in Spain that used a food frequency questionnaire (FFQ) to measure DD. Nutritional insufficiency was defined as deficiency in at least four of 17 nutrients below two-thirds of the Dietary Reference Intakes (DRIs). Results showed that older adults with the lowest DD had a significantly higher risk of inadequate nutrient intake compared to those with the highest DD (OR = 28.56, *p* < 0.05). Research confirms that DD serves as a protective factor against cardiovascular disease risk and can also function as an intervention strategy tool for lifestyle management in older adults with existing cardiovascular disease (35). Therefore, controlling malnutrition in the elderly depends on boosting DD. Since only cross-sectional studies (36) have looked at the relationship between DD and nutritional status in CHF patients, intervention studies are needed to confirm the causal relationship. In order to provide data for clinical practice, this study examines how DD intervention affects the nutritional status of elderly CHF patients who are able to eat orally.

Unlike conventional low-sodium or general nutrition guidance for heart failure, this intervention incorporated a structured, tiered dietary diversification model based on the Dietary Diversity Score (DDS), enabling personalized and quantifiable monitoring of food group balance. This patient-centered, behavior-oriented framework integrates clinical nutrition assessment (MNA-HF) with practical dietary education, representing an innovative approach for improving nutritional quality and self-management in elderly CHF patients.

## 2 Materials and methods

### 2.1 Establishing a dietary diversification intervention program for elderly patients with chronic heart failure who can eat by mouth

#### 2.1.1 Establish a research team

The research team consists of eight members: one head nurse in cardiovascular medicine overseeing project guidance, two specialists responsible for disease diagnosis and treatment, one nutritionist reviewing and modifying dietary management plans, three senior registered nurses handling patient education and follow-up management, and one research nurse primarily coordinating overall project operations.

#### 2.1.2 Development of dietary diversification intervention programs

This study retrieved literature from databases including CNKI, Wanfang, VIP, CBM, PubMed, Ovid, Web of Science databases using keywords such as “Heart Failure,” “nutrition,” “Dietary diversity,” and “DDS.” Concurrently, domestic and international guideline websites were searched, including the Scottish Intercollegiate Guidelines Network (SIGN), the National Institute for Health and Care Excellence (NICE), The Agency for Healthcare Research and Quality’s (AHRQ) Clinical Practice Guidelines Database, and the MedlinePlus Clinical Guidelines website. This research aimed to understand domestic and international dietary recommendations for elderly CHF patients consuming food orally, forming a preliminary draft of the dietary diversity intervention plan. Subsequently, 10 experts were invited to refine the intervention plan through an expert meeting method, resulting in the final dietary diversity intervention plan (Table 1).

**Table 1:** Intervention protocols for different dehydration levels in elderly CHF patients receiving oral feeding.

Time period	Intervention topic	Specific measures
Admission 1-2 days	Nutritional status assessment	The Modified Nutritional Assessment for Heart Failure (MNA-HF) was used to assess the nutritional status of elderly CHF patients receiving oral nutrition.
	Dietary diversity assessment	A food frequency questionnaire (FFQ) was used to collect data on the weekly intake of food items consumed orally by elderly CHF patients. Concurrently, the Dietary Diversity Score (DDS9) was administered to assess dietary diversity levels prior to hospitalization.
	Gastrointestinal function assessment	Assess gastrointestinal congestion symptoms in elderly CHF patients receiving oral feeding, such as abdominal distension, nausea, vomiting, and loss of appetite.
Admission 2-3 days	Patient dietary guidance	(1) Advise family members to prioritize preparing soft-textured, easily digestible foods with high energy and nutrient density, such as minced meat congee, minced meat, meatballs, egg custard, wontons, and soft rice. Avoid preparing greasy, overly salty, excessively sweet, or spicy foods.
		(2) Inform patients of the importance of eating for recovery, encouraging them to eat smaller, more frequent meals (4–6 times/day).
Admission 4-5 days	Dietary diversity knowledge presentation	After the attending physician assessed the patient’s condition as stable, the research team conducted a face-to-face PowerPoint presentation to educate the patient on dietary diversity. The main content was as follows:
		(1) The concept of dietary diversity;
		(2) Benefits of dietary diversity for elderly CHF patients eating by mouth;
		(3) Food groups included in dietary diversity, nutritional components of various foods, and common food items;
		(4) Rational combination of dietary diversity;
		(5) Intake levels for various food categories.
Admission 5-6 days	Dietary diversity classification guidance	Based on pre-admission dietary diversity assessment results, combined with the patient’s economic status and dependency situation, individualized dietary diversity recommendations are provided:
		(1) Elderly CHF patients with inadequate dietary diversity (DDS 1–3 points) who are able to eat orally Diet consists solely of three categories: grains, vegetables, and oils, with limited variety and particularly lacking protein-rich foods. Recommended dietary diversification involves adding eggs, soy products, lean meats, and common fruits to the existing diet. Where feasible, incorporate dairy products and fish while appropriately controlling oil and salt intake.
		① Eggs: Rich in nutrients with high protein bioavailability, eggs are among the most ideal sources of high-quality protein in the diet.
		How much: 1 egg per day or an equivalent amount of other egg products.
		How to eat: Consume eggs boiled, in egg custard, scrambled with tomatoes, fried, or in egg drop soup. Avoid consuming raw eggs.
		② Soy products: Rich in protein, calcium, potassium, vitamin E, and unsaturated fatty acids, they are a high-quality protein source.
		How much: 15 g–25 g/day (4–6 servings/week, approximately 2 ounces).
		How to eat: Soy milk, tofu pudding, tofu, dried tofu, dried bean curd sticks, bean sprouts, etc. Various soy products can be combined with other foods in daily meals.
		③ Lean meat: Rich in protein and containing various essential micronutrients for the human body, including lean cuts from pork, beef, chicken, duck, and other poultry.
		How much: 40 g–50 g/day, 300 g–500 g/week.
		How to prepare: Combine with other vegetables in dishes such as minced meat, meatballs, meat filling, shredded meat, or sliced meat.
		④ Fruits: Rich in nutrients like vitamin C, potassium, and dietary fiber, they stimulate appetite and relieve constipation.
		How much: 200 g–300 g/day (about the size of one medium apple).
		How to eat: Choose soft-textured fruits like citrus, dragon fruit, kiwi, peaches, apples, and bananas. For harder fruits, cut into small pieces or cook until soft. Avoid juicing whenever possible.
		⑤ Dairy products and fish: Rich in protein and essential minerals for the human body.
		How much: Senior formula milk powder 30 g/day, fish 2 times/week.
		How to eat: Choose liquid milk if conditions permit; opt for senior formula milk powder if conditions are limited. For fish, select steamed or boiled options (be mindful of bones and eat slowly).
		⑥ Grains: Increase intake of whole grains and legumes rich in dietary fiber and vitamin B12.
		⑦ Vegetables: Rich in vitamins.
		How much to eat: At least 300 g of fresh vegetables daily, with half being dark-colored varieties. Ensure vegetables are included in every meal.
		How to eat: Cut into small pieces or use as filling, and combine with other ingredients to prepare dishes.
		⑧ Salt and oil
		Salt: Sodium intake is not strictly restricted for elderly CHF patients with mild or stable oral intake. For patients with volume overload, sodium intake should not exceed 2 g/day.
		Oil: 25 g–30 g/day, using vegetable oils to partially replace animal fats.
		(2) Dietary Diversity for Elderly CHF Patients with moderate oral intake (DDS 4–6 points) Primary food groups consumed include cereals, vegetables, meat, and fats/oils—four categories—but protein-rich food intake remains limited. To enhance dietary diversity, it is recommended to add eggs, soy products, seafood, dairy products, and fruits to the existing diet, along with moderate lean meat consumption.
		① Eggs: Rich in nutrients with high protein bioavailability, eggs are among the most ideal sources of high-quality protein in the diet.
		How much: 1 egg per day or an equivalent amount of other egg products.
		How to eat: Consume eggs boiled, in egg custard, scrambled with tomatoes, fried, or in egg drop soup. Avoid consuming raw eggs.
		② Soy products: Rich in protein, calcium, potassium, vitamin E, and unsaturated fatty acids, they are a high-quality protein source.
		How much: 15 g–25 g/day (4–6 servings/week, approximately 2 ounces).
		How to eat: Soy milk, tofu pudding, tofu, dried tofu, dried bean curd sticks, bean sprouts, etc. Various soy products can be combined with other foods in daily meals.
		③ Seafood: Rich in high-quality protein, lipids, vitamins, and minerals. It is particularly high in protein, low in carbohydrates, and abundant in unsaturated essential fatty acids.
		How much to eat: Consume twice weekly or 300 g–500 g per week.
		How to prepare: Boiling, steaming, or stir-frying are recommended methods that cause relatively minimal nutrient loss.
		④ Dairy products: A high-quality protein rich in nutrients, with a balanced composition and easily digestible.
		How much: 300 mL/day of liquid milk.
		How to consume: Drink at breakfast or in the evening. Overweight or obese individuals should choose skim or low-fat milk. Those with lactose intolerance may opt for fermented yogurt.
		⑤ Fruits: Rich in nutrients like vitamin C, potassium, and dietary fiber, they stimulate appetite, aid digestion, and relieve constipation.
		How much: 200 g–300 g/day, approximately one medium-sized apple.
		How to eat: Choose soft-textured fruits like dragon fruit, kiwi, peaches, citrus, apples, and bananas. For harder fruits, cut into small pieces or cook until soft. Avoid juicing whenever possible.
		⑥ Lean meat: Rich in protein and containing various essential micronutrients for the human body, including lean cuts from pork, beef, chicken, duck, and other poultry.
		How much: 40 g–50 g/day, 300 g–500 g/week.
		How to prepare: Combine with other vegetables in dishes such as minced meat, meatballs, meat filling, shredded meat, or sliced meat.
		⑦ Vegetables: Rich in vitamins.
		How much: 300 g/day of fresh vegetables, with half being dark-colored varieties. Include vegetables with every meal.
		How to prepare: Cut into small pieces or use as stuffing. Can be combined with other foods in recipes.
		⑧ Grains: Limit refined grains and increase whole grains and legumes rich in dietary fiber and vitamin B12.
		⑨ Salt and oil
		Salt: Sodium intake is not strictly restricted for elderly CHF patients with mild or stable oral intake. For patients with volume overload, sodium intake should not exceed 2 g/day.
		Oil: 25 g–30 g/day, using vegetable oils to partially replace animal fats.
		(3) Elderly CHF patients with adequate dietary diversity (DDS 7–9 points)
		The food groups consumed are relatively diverse, but intake of dairy products and seafood remains insufficient. Dietary diversity recommendations suggest increasing consumption of dairy products and seafood based on the existing diet.
		① Dairy products: Rich in nutrients, with a balanced composition and easily digestible high-quality protein.
		How much: 300 mL/day of liquid milk.
		How to consume: Drink at breakfast or in the evening. Overweight or obese individuals should choose skim milk or low-fat milk. Those with lactose intolerance may opt for fermented yogurt.
		② Seafood: Rich in high-quality protein, lipids, vitamins, and minerals. It features a relatively high protein content, low carbohydrates, and fats primarily composed of unsaturated essential fatty acids.
		How much to eat: Consume fish twice weekly or 300 g–500 g per week.
		How to prepare: Opt for forms like fish balls or shrimp paste, which enhance digestibility and nutrient absorption.
	Dietary guidance for common comorbidities	(1) Diabetes
		① Adjust meal order: Vegetables—Meat—Starchy foods to reduce postprandial blood glucose response.
	Promoting dietary diversity	② Increase vegetable intake at every meal (over 500 g daily). Pair each meal with protein-rich foods; even diabetic nephropathy patients should consume 0.8 g protein/kg body weight daily.
		③ Suitable fruits: apples, pears, peaches, apricots, plums, cherries, grapes, citrus fruits, and pomelos. Consume watermelon, pineapple, mango, and kiwi in moderation. Limit bananas and fresh jujubes. Ensure daily fruit intake of 100 g–200 g. Fruits should not be consumed with main meals, but rather as snacks between meals or before bedtime.
		(2) Hypertension
		Consume 1 kg of fresh vegetables and 500 g of fruit daily, maintain a low-salt diet <5 g. For obese patients, gradually manage and maintain a healthy weight, actively control blood pressure, quit smoking, and limit alcohol consumption.
		(3) Hyperlipidemia
		Prioritize whole grains, soy products, and okra. For oils, choose olive oil, tea seed oil, canola oil, flaxseed oil, and perilla oil. Increase consumption of fish and shellfish, and drink more green tea. Limit eggs to no more than one per day or 3–6 per week. Restrict animal offal.
		(4) Hyperuricemia or gout
		① During gout flare-ups, avoid high-purine foods and opt for low-purine options like grains, tubers, and vegetables. For asymptomatic hyperuricemia, moderate-purine foods such as fruits, eggs, dairy, and soy products are permissible.
		② Avoid consuming large quantities of meat, fish, or shrimp together with alcohol, as this may trigger gout attacks.
		③ Limit intake of sweets or sugary beverages and minimize consumption of fruit juices (including juice drinks and fresh-squeezed juices). Individuals with normal uric acid levels should consume approximately 200 g of fresh fruit per day.
		④ Patients with hyperuricemia may consume 2 eggs and 500 mL of milk (preferably skim milk) daily while avoiding meat, fish, and shellfish.
		⑤ Soy products may be consumed normally, in moderation, or avoided entirely based on the patient’s uric acid levels, frequency of gout attacks, and intake of other purine-rich foods.
Before discharge from the hospital	Individualized guidance	(1) Energy requirements: carbohydrates primarily from grains and tubers (50–65%), fats mainly from vegetable oils and animal fats (20–30%), proteins mainly from livestock, poultry, meat, fish, and soybeans (10–15%). Energy requirement is 25–30 kcal/kg/day.
		(2) Provide face-to-face, individualized dietary diversity guidance based on the patient’s most recent nutritional biochemical test results and energy requirements.
		(3) Use the teach-back method to assess the patient’s understanding level, promptly correcting any areas of weakness or misconceptions.
		(4) Distribute a corresponding dietary diversity health handbook.
After discharge from the hospital	Maintaining dietary diversity	(1) Conduct telephone interventions every 2 weeks for 2 months following discharge.
		(2) Assess patients’ home dietary habits and dietary diversity, and provide detailed explanations addressing common dietary misconceptions, including the following:
		(a) Does a light diet for heart failure patients mean plain rice porridge with vegetables? How can one achieve a light diet?
		(b) How can elderly patients with chronic heart failure reduce salt intake in daily life?
		(c) Are fruits off-limits for elderly patients with chronic heart failure and diabetes?
		(d) Can elderly patients with chronic heart failure and hyperuricemia, or those with a history of gout, consume soy products?
		(e) Are various meat broths truly nutritious?
		(f) Why are milk and eggs so important?
		(g) Can elderly heart failure patients eat so-called “trigger foods” like chicken, duck, or fish?

### 2.2 Implementation of the dietary diversity intervention program

#### 2.2.1 Study design and participants

A quasi-experimental study with a non-randomized, non-concurrent control design was used. This design was selected to prevent intervention contamination between groups residing in the same ward environments. To minimize potential temporal bias, the medical team, nursing protocols, and general ward environment remained consistent throughout the study period (July–October 2022). Convenience sampling was employed to select study subjects: elderly CHF patients admitted to the Cardiovascular Department of a Grade III Class A hospital in Zunyi city, Guizhou province, between July and October 2022, who were able to eat orally, were conveniently selected based on inclusion/exclusion criteria. A non-concurrent control grouping was simultaneously applied: patients admitted between July and August 2022 were designated as the control group, while those admitted between September and October 2022 formed the intervention group. The following are the inclusion criteria: (1) Meets the diagnostic criteria for CHF in the Chinese Guidelines for Diagnosis and Treatment of Heart Failure 2018 (1); (2) New York Heart Association (NYHA) functional class II–IV; (3) Age≥60 years with oral intake; (4) Ability to communicate verbally and sign informed consent. Exclusion criteria were as follows: (1) Receiving nasogastric tube feeding or parenteral nutrition support; (2) Concurrent severe hepatic or renal impairment, malignancy, or abdominal surgery within the past 3 months; (3) Presence of severe cognitive impairment or sensory deficits. The sample size was calculated using the formula for two-sample mean comparison based on the primary outcome variable, the Total Dietary Diversity Score (DDS): *N*_1_ = *N*_2_ = 2[*σ* (*t*_∂_ + *t_β_*)/(*μ*_1_ − *μ*_2_)]^2^. Where *n* is the required sample size per group, *σ*^2^ represents the estimated variance, and *δ* denotes the expected mean difference between the intervention and control groups. According to pilot data collected from 20 elderly CHF patients before the main study, the mean difference in Total DDS between groups was approximately 0.9, with a pooled standard deviation of 1.5—corresponding to a medium effect size (Cohen’s *d* = 0.6). Assuming a two-tailed *α* = 0.05 and 80% power (*β* = 0.20), the required minimum sample size was calculated as 33 participants per group (total = 66). Considering an anticipated 20% attrition rate during the two-month follow-up, the final target sample was increased to 88 participants (44 in each group) to ensure adequate statistical power to detect clinically meaningful differences in dietary diversity.

#### 2.2.2 Intervention

Studies show that traditional face-to-face education strategies do not produce better results than online nutrition health education for older CHF patients (37). For the intervention and control groups, this study mostly used in-hospital, face-to-face, one-on-one explanations because of the subjects’ low educational background and lack of familiarity with smart technologies. Interventions outside of the hospital mostly consisted of phone conversations with family WeChat groups added for clarification. According to a University College London research, it takes an average of 66 days to acquire a habit (38). As a result, the intervention period in this study for both groups lasted from 1 to 2 days following admission to 2 months following release.

##### 2.2.2.1 Control group

Following admission, the control group was mainly given standard health education, which included recommendations for patients to eat smaller, more often meals and to follow diets that are easy to digest and low in fat and salt.

##### 2.2.2.2 Intervention group

The intervention group implemented different daily dose (DD) intervention protocols for elderly CHF patients receiving oral feeding, building upon the control group’s approach. To ensure reproducibility and methodological transparency, the dietary diversification intervention was structured into clearly defined core components with standardized delivery and intensity. The intervention consisted of five key elements: (1) baseline assessment using the Dietary Diversity Score (DDS9) and the Mini Nutritional Assessment for Heart Failure (MNA-HF); (2) individualized dietary guidance stratified by DDS level (low, moderate, or adequate diversity), integrated with comorbidity-specific nutrition advice; (3) structured educational sessions using standardized PowerPoint materials and visual handouts during hospitalization; (4) reinforcement through biweekly follow-up phone calls for 2 months after discharge to monitor adherence and provide corrective feedback; and (5) fidelity assurance through periodic supervision by trained senior nurses and cross-checking of intervention logs.

The intervention intensity was moderate, with in-hospital sessions lasting 30–40 min and follow-up calls averaging 15 min per contact. Fidelity was assessed by adherence checklists and independent review of intervention records to confirm consistency across participants and educators. This structured and supervised design enhances both internal validity and replicability in similar clinical settings.

### 2.3 Quality control

The following methods are used to control the quality of research: ① Instruction on pertinent nutrition for research team members: Members of the study team were asked to participate in online training courses led by registered dietitians from the Clinical Nutrition Department. The seven major nutrients, food and nutrition, geriatric nutritional needs, and dietary management for prevalent diseases in the elderly were among the subjects discussed in these sessions. There were five sessions, each lasting roughly 1-2 hours. After finishing the training, each team member took an evaluation, and they were only allowed to take part in the intervention if they passed. ② Training in intervention content: Team members received thorough instructions on the intervention protocol, steps, communication strategies, and safety measures, including how to treat patients with respect and calmly answer their concerns. Only eligible members were allowed to participate in interventions, and comprehension levels were assessed by post-training case analysis exams. To increase acceptance and the efficacy of teaching, all interventions were carried out when patient conditions were stable. Before starting the study, team members give patients a detailed explanation of the initiative and gage their interest in taking part. While patiently answering all questions, communicate facts using clear language and striking examples. Get patient and family contact information to reduce post-discharge loss to follow-up. Moreover, accurately record dropouts and missing cases in the study data and examine the reasons for loss to follow-up. Patients were questioned about their application of dietary diversity, given recommendations accordingly, and had their queries answered during biweekly telephone interactions. Data were double-checked for accuracy following questionnaire collection and then entered into the SPSS 29.0 database. Any logical errors or missing information were quickly fixed and added to. After then, data were combined and rigorously examined using statistical techniques.

### 2.4 Outcome measures

DD assessment encompasses simple counting methods and composite index methods. Simple counting methods only tally food types without considering nutrient intake; composite index methods account for both food types and nutrient intake, Simple counting methods include the Dietary Diversity Score (DDS) and Food Variety Score (FVS) (39). DDS imposes minimal requirements on respondents, making it suitable for rural populations and the elderly (40). It falls into one of three categories: DDS5, DDS9, or DDS28, depending on how many food groups were examined. Research comparing the efficacy of DDS5, DDS9, DDS28, and FVS in determining nutritional adequacy shows that DDS9 is the most widely used and provides the most efficiency and effectiveness in comprehensive evaluation. The DDS9 classifies foods into nine classes, including cereals, tubers and pulses, vegetables, fruits, poultry and animal meat, eggs, dairy and dairy products, soybeans, fish, and oils and fats. It is based on the food pyramid included in the Chinese Dietary Guidelines. With a minimum score of 0 and a maximum score of 9, one point is awarded for each DDS9 category consumed. Both the frequency and the amount of food consumed are not recorded, nor is intake within the same category reported more than once. Three levels of DDS9 scores are distinguished: inadequate dietary diversity is indicated by DDS1-3, moderate dietary diversity is indicated by DDS4-6, and appropriate dietary diversity is indicated by DDS7-9 (41).

Food Frequency Questionnaire: The Food Frequency Questionnaire (FFQ) and 24-h dietary recall are the two main dietary data collection tools used for DDS. The 24-h dietary recall has limits in terms of survey duration and demographic applicability, but it produces extremely accurate dietary data with little memory bias. A popular dietary assessment technique in nutritional epidemiology research is the FFQ. They can show participants’ eating patterns as well as the quantity, frequency, and availability of particular meals over time. FFQs have several benefits, including affordability, simplicity, and high response rates. Depending on the target population, they can be classified as qualitative, quantitative, or semi-quantitative. The participants in this study were elderly Guizhou Province CHF patients. A qualitative FFQ was used in accordance with the DDS9 evaluation requirements in order to document the frequency of consuming different food kinds over a certain time period without quantifying amounts. Data on food types consumed by patients during a one-week period (42) were gathered using the FFQ created for the 2010 China National Nutrition Survey (39). The qualitative FFQ was selected primarily to ensure feasibility and reliability among elderly CHF patients, many of whom had limited literacy and memory capacity. Quantitative methods such as 24-h dietary recall or weighed food records were deemed impractical in this population, as they require precise portion estimation and frequent documentation. The qualitative FFQ, by focusing on food types and frequency rather than amounts, allowed participants to report their dietary patterns more accurately within their cognitive and physical capabilities, thereby improving response validity and reducing recall burden. However, it cannot directly estimate nutrient quantities, which is acknowledged as a methodological limitation discussed below.

The Mini-Nutrition Assessment Special for Heart Failure (MNA-HF) is a nutritional assessment tool that was developed by Lin et al. (43). By incorporating characteristics of heart failure. Validity and reliability validation showed that the modified MNA-HF achieved a Cronbach’s alpha of 0.710, higher than the MNA’s 0.613, and exhibited greater authenticity in assessing the nutritional status of HF patients compared to the MNA (43). The MNA-HF scale consists of four dimensions: anthropometry, dietary intake assessment, overall assessment, and self-assessment. Scores range from 0 to 29 points; a score of ≥22 indicates adequate nutritional status; 16–21 points suggests malnutrition risk; and <16 points confirms malnutrition. The tool’s benefits include simplicity, convenience, cost-effectiveness, and non-invasiveness (43).

### 2.5 Data analysis

Data entry was performed using Excel, and statistical analysis was conducted using SPSS 27.0 software. Continuous variables were tested for normality using the Kolmogorov–Smirnov test. Variables conforming to a normal distribution (including BMI, DDS, and MNA-HF) were expressed as mean ± standard deviation. Differences between the intervention and control groups at baseline, 1 month, and 2 months were analyzed using independent samples *t*-tests. To quantify the magnitude of differences, Cohen’s *d* was calculated as the effect size, along with its 95% confidence interval (CI). Effect sizes were interpreted as small (*d* = 0.2), medium (*d* = 0.5), and large (*d* = 0.8). Categorical data were expressed as frequencies and percentages [*n* (%)]. Comparisons between groups were performed using the Chi-square test or Fisher’s exact test (used when theoretical frequencies were less than 5, addressing small sample size limitations). Effect sizes for categorical variables were reported using Phi coefficient (for 2 × 2 tables) or Cramer’s *V* (for tables larger than 2 × 2). Trends in outcome measures across the three time points were analyzed using repeated measures ANOVA. The effect size for ANOVA was reported as partial eta squared (partial *η*^2^), where 0.01, 0.06, and 0.14 indicate small, medium, and large effects, respectively. Paired samples *t*-tests were used to compare BMI differences within groups (pre- vs. post-intervention), and independent *t*-tests were used to compare the difference scores (change values) between groups. A two-tailed *p*-value <0.05 was considered statistically significant.

Analyses were conducted on a per-protocol (PP) basis, including only participants who completed the full two-month follow-up. Missing data resulting from deaths or loss to follow-up were not imputed, but these cases were documented and analyzed descriptively to assess potential bias. The per-protocol approach was chosen to ensure that the observed effects directly reflected the impact of full exposure to the dietary diversification intervention.

## 3 Results

For this trial, 88 elderly CHF patients who were able to eat orally were first enrolled; 44 were assigned to the intervention group and 44 to the control group. By the end of the first month following release, two deaths had occurred in the intervention group, and by the end of the second month, two more had occurred. In the control group, eight patients were lost to follow-up; three of them died by the end of the first month following release, and one by the end of the second. Ultimately, 80 patients completed the entire intervention, 40 of whom were in the intervention group and 40 of whom were in the control group.

### 3.1 Comparison of two sets of baseline data

The two groups were comparable with no statistically significant differences in age, gender, marital status, education level, occupation, income, food expenses, medical insurance, lifestyle habits (smoking, alcohol), or clinical characteristics (heart function, comorbidities, etiology) (*p* > 0.05). Effect size analysis confirmed the balance between groups, with all variables showing small or negligible effect sizes (Cohen’s *d* < 0.2 for age; Phi or Cramer’s *V* < 0.3 for categorical variables) (see Table 2).

**Table 2:** Baseline data for both patient groups prior to intervention.

Variable	Control group (*n* = 40)	Intervention group (*n* = 40)	Statistic	*p*-value	Effect size
Age (years), mean ± SD	75.15 ± 7.12	74.25 ± 6.31	*t* = 0.60	0.552	*d* = 0.13
Gender, *n* (%)			*χ*^2^ = 1.2	0.262	Phi = 0.12
Male	24 (55.8%)	19 (44.2%)			
Female	16 (43.2%)	21 (56.8%)			
Marital status, *n* (%)			*χ*^2^ = 0.83	0.722	*V* = 0.10
Married	24 (48.0%)	26 (52.0%)			
Single	1 (33.3%)	2 (66.7%)			
Widowed	15 (55.6%)	12 (44.4%)			
Educational level, *n* (%)			*χ*^2^ = 7.75	0.052	*V* = 0.31
Illiterate	15 (51.7%)	14 (48.3%)			
Primary school or below	13 (76.5%)	4 (23.5%)			
Junior high school	8 (34.8%)	15 (65.2%)			
Senior high school	4 (36.4%)	7 (63.6%)			
Nature of the occupation			*χ*^2^ = 0.00	>0.999	Phi = 0.00
Mental labor	13 (50.0%)	13 (50.0%)			
Physical labor	27 (50.0%)	27 (50.0%)			
Monthly income (RMB)			*χ*^2^ = 0.13	0.936	*V* = 0.04
<2,500	24 (49.0%)	25 (51.0%)			
2,500–4,999	11 (50.0%)	11 (50.0%)			
≥5,000	5 (55.6%)	4 (44.4%)			
Monthly food expenses (RMB)			*χ*^2^ = 0.30	0.862	*V* = 0.06
<2,500	28 (51.9%)	26 (48.1%)			
2,500–4,999	8 (44.4%)	10 (55.6%)			
≥5,000	4 (50.0%)	4 (50.0%)			
Types of medical insurance			*χ*^2^ = 0.24	0.626	Phi = 0.05
Rural/urban medical insurance	29 (51.8%)	27 (48.2%)			
Employee medical insurance	11 (45.8%)	13 (54.2%)			
Smoke			*χ*^2^ = 3.41	0.065	Phi = 0.21
Yes	19 (63.3%)	11 (36.7%)			
No	21 (42.0%)	29 (58.0%)			
Drinking alcohol			*χ*^2^ = 1.53	0.217	Phi = 0.14
Yes	14 (60.9%)	9 (39.1%)			
No	26 (45.6%)	31 (54.4%)			
Living alone			*χ*^2^ = 0.00	>0.999	Phi = 0.00
Yes	7 (50.0%)	7 (50.0%)			
No	33 (0.0%)	33 (50.0%)			
Chewing ability			*χ*^2^ = 4.61	0.100	*V* = 0.24
Good	6 (54.5%)	5 (45.5%)			
Average	29 (56.9%)	22 (43.1%)			
Poor	5 (27.78%)	13 (72.22%)			
Dosage (types)			*χ*^2^ = 1.40	0.237	Phi = 0.13
<5	11(40.7%)	16(59.3%)			
≥5	29(54.7%)	24(45.3%)			
Heart failure duration (months)			*χ*^2^ = 1.57	0.210	Phi = 0.14
<60	32 (47.1%)	36 (52.9%)			
≥60	8 (66.7%)	4 (33.3%)			
Comorbidities (types)			*χ*^2^ = 1.46	0.228	Phi = 0.14
<5	25 (45.5%)	30 (54.5%)			
≥5	15 (60.0%)	10 (40.0%)			
Heart function classification			*χ*^2^ = 2.57	0.323	*V* = 0.18
II	8 (36.4%)	14 (63.6%)			
III	28 (53.8%)	24 (46.2%)			
IV	4 (66.7%)	2 (33.3%)			
Etiology			*χ*^2^ = 1.47	0.744	*V* = 0.14
Coronary heart disease	28 (54.9%)	23 (45.1%)			
Valvular heart disease	4 (40.0%)	6 (60.0%)			
Hypertension	2 (40.0%)	3 (60.0%)			
Other	6 (42.9%)	8 (57.1%)			

Data are presented as mean ± SD or *n* (%); Effect size: Reported as Cohen’s *d* for continuous variables and Phi (for 2 × 2) or Cramer’s *V* (for >2 categories) for categorical variables; Statistic refers to *t*-value (for Age) or *χ*^2^ value (for others). *p* > 0.05 indicates no significant difference at baseline.

### 3.2 Comparison of DD and nutritional status between the two groups before intervention

At baseline, there were no statistically significant differences between the two groups in Total DDS, BMI, MNA-HF scores, or most specific food group scores (*p* > 0.05), indicating comparable nutritional status prior to the intervention. The only exception was Grains DDS, which was significantly higher in the intervention group (*t* = −2.08, *p* = 0.041), with a medium effect size (Cohen’s *d* = 0.46). All other variables showed small effect sizes (*d* < 0.2), further supporting baseline comparability (see Table 3).

**Table 3:** Comparison of DD and nutritional status between the two groups before intervention ( x_±s ).

Variable	Control group (*n* = 40)	Intervention group (*n* = 40)	*t*	*p*	Cohen’s *d*	95% CI (*d*)
Total DDS	5.63 ± 1.33	5.70 ± 1.24	−0.26	0.796	0.056	−0.37 ~ 0.48
BMI	22.48 ± 3.73	22.45 ± 3.43	0.03	0.978	0.008	−0.37 ~ 0.39
MNA-HF	21.09 ± 4.34	21.54 ± 3.67	−0.50	0.618	0.119	−0.30 ~ 0.54
Grains DDS	2.18 ± 1.01	2.60 ± 0.81	−2.08	0.041	0.463	0.02 ~ 0.88
Legumes DDS	0.78 ± 1.19	0.75 ± 1.06	0.10	0.921	0.022	−0.34 ~ 0.38
Vegetables DDS	4.13 ± 1.87	4.43 ± 2.17	−0.66	0.510	0.147	−0.27 ~ 0.55
Fruits DDS	1.03 ± 1.27	1.23 ± 1.58	−0.63	0.534	0.141	−0.28 ~ 0.56
Dairy products DDS	0.43 ± 0.50	0.35 ± 0.48	0.68	0.497	0.152	−0.26 ~ 0.57
Meat DDS	0.95 ± 0.39	0.93 ± 0.62	0.22	0.829	0.050	−0.36 ~ 0.47
Fish DDS	0.10 ± 0.30	0.15 ± 0.43	−0.60	0.548	0.136	−0.29 ~ 0.55
Eggs DDS	0.38 ± 0.49	0.55 ± 0.50	−1.57	0.119	0.351	−0.09 ~ 0.79
Oils and fats DDS	1.60 ± 0.50	1.68 ± 0.53	−0.66	0.514	0.147	−0.28 ~ 0.58

DDS, Dietary Diversity Score; BMI, Body Mass Index; MNA-HF, Malnutrition Assessment Tool for the Elderly; Cohen’s *d* is reported as the absolute effect size of the difference, with 0.2, 0.5, and 0.8 representing small, medium, and large effects, respectively.

### 3.3 Comparison of DD and nutritional status between the two groups after 1 month of intervention

After 1 month of intervention, the intervention group demonstrated significantly higher scores than the control group for Total DDS (*t* = −6.03, *p* < 0.001), with a large effect size (Cohen’s *d* = 1.35). Significant improvements were also observed in specific food categories. Fish DDS showed the largest effect (*d* = 0.98, *p* < 0.001), followed by Fruits (*d* = 0.68, *p* = 0.003), Eggs (*d* = 0.63, *p* = 0.006), Legumes (*d* = 0.52, *p* = 0.022), and Grains (*d* = 0.49, *p* = 0.031). However, no statistically significant differences were observed between groups for BMI, MNA-HF, vegetables, dairy products, meat, or oils (*p* > 0.05), with effect sizes remaining small (see Table 4).

**Table 4:** Comparison of DD and nutritional status between the two groups after 1 month of intervention.

Variable	Control group (*n* = 40)	Intervention group (*n* = 40)	*t*	*p*	Cohen’s *d*	95% CI (*d*)
Total DDS	6.30 ± 1.14	7.68 ± 0.89	−6.03	**<0.001**	1.35	0.86 ~ 1.84
BMI	22.84 ± 3.45	23.02 ± 2.93	−0.26	0.795	0.06	−0.38 ~ 0.50
MNA-HF	21.86 ± 3.81	22.65 ± 2.87	−1.04	0.300	0.23	−0.21 ~ 0.67
Grains DDS	2.18 ± 1.01	2.63 ± 0.81	−2.20	**0.031**	0.49	0.05 ~ 0.94
Legumes DDS	0.78 ± 1.17	1.38 ± 1.13	−2.34	**0.022**	0.52	0.08 ~ 0.97
Vegetables DDS	4.15 ± 1.82	4.78 ± 1.70	−1.59	0.117	0.36	−0.09 ~ 0.80
Fruits DDS	1.63 ± 1.17	2.38 ± 1.03	−3.04	**0.003**	0.68	0.23 ~ 1.13
Dairy products DDS	0.43 ± 0.50	0.55 ± 0.50	−1.11	0.269	0.25	−0.19 ~ 0.69
Meat DDS	0.93 ± 0.42	1.08 ± 0.53	−1.41	0.161	0.32	−0.13 ~ 0.76
Fish DDS	0.08 ± 0.27	0.50 ± 0.55	−4.37	<**0.001**	0.98	0.51 ~ 1.44
Eggs DDS	0.78 ± 0.42	0.98 ± 0.16	−2.80	**0.006**	0.63	0.18 ~ 1.08
Oils and fats DDS	1.60 ± 0.50	1.73 ± 0.45	−1.18	0.243	0.26	−0.18 ~ 0.70

DDS, Dietary Diversity Score; BMI, Body Mass Index; MNA-HF, Mini Nutritional Assessment for Heart Failure; Cohen’s *d* is reported as the absolute effect size of the difference, with 0.2, 0.5, and 0.8 representing small, medium, and large effects, respectively. Bold values indicate statistically significant results at *p* < 0.05.

### 3.4 Comparison of DD and nutritional status between the two groups after 2 months of intervention

After 2 months, the intervention effects became more pronounced. The intervention group exhibited significantly higher scores than the control group for Total DDS (*t* = −7.68, *p* < 0.001), with a very large effect size (Cohen’s *d* = 1.72). Notably, the MNA-HF score was significantly higher in the intervention group compared to the control group (24.51 ± 2.27 vs. 23.00 ± 3.06; *t* = −2.52, *p* = 0.014), representing a medium effect size (Cohen’s *d* = 0.56). Regarding specific food groups, significant differences were found in Fish (*d* = 1.32), Vegetables (*d* = 0.79), Fruits (*d* = 0.73), Eggs (*d* = 0.58), Meat (*d* = 0.55), and Grains (*d* = 0.49) (all *p* < 0.05). No statistically significant differences were observed for BMI, dairy products, or oils (*p* > 0.05) (see Table 5).

**Table 5:** Comparison of DD and nutritional status between the two groups after 2 months of intervention

Variable	Control group (*n* = 40)	Intervention group (*n* = 40)	*t*	*p*	Cohen’s *d*	95% CI (*d*)
Total DDS	6.40 ± 1.01	7.95 ± 0.78	−7.68	**<0.001**	**1.72**	1.23 ~ 2.21
BMI	22.94 ± 3.29	23.30 ± 2.80	−0.52	0.604	0.12	−0.33 ~ 0.56
MNA-HF	23.00 ± 3.06	24.51 ± 2.27	−2.52	**0.014**	0.56	0.11 ~ 1.00
Grains DDS	2.18 ± 1.01	2.63 ± 0.81	−2.20	**0.031**	0.49	0.05 ~ 0.94
Legumes DDS	4.15 ± 1.82	4.83 ± 1.71	−1.71	0.091	0.38	−0.06 ~ 0.81
Vegetables DDS	1.63 ± 1.17	2.48 ± 0.96	−3.55	**0.001**	0.79	0.33 ~ 1.24
Fruits DDS	0.80 ± 1.16	1.65 ± 1.14	−3.30	**0.001**	0.73	0.27 ~ 1.18
Dairy products DDS	0.43 ± 0.50	0.55 ± 0.50	−1.11	0.269	0.25	−0.19 ~ 0.69
Meat DDS	0.93 ± 0.42	1.20 ± 0.56	−2.48	**0.015**	0.55	0.10 ~ 0.98
Fish DDS	0.10 ± 0.30	0.8 ± 0.69	−5.90	**<0.001**	1.32	0.85 ~ 1.79
Eggs DDS	0.85 ± 0.36	1.00 ± 0.00	−2.62	**0.010**	0.58	0.13 ~ 1.02
Oils and fats DDS	1.60 ± 0.50	1.73 ± 0.45	−1.18	0.243	0.26	−0.18 ~ 0.70

DDS, Dietary Diversity Score; BMI, Body Mass Index; MNA-HF, Mini Nutritional Assessment for Heart Failure; Cohen’s *d* is reported as the absolute effect size of the difference, with 0.2, 0.5, and 0.8 representing small, medium, and large effects, respectively. Bold values indicate statistically significant results at *p* < 0.05.

### 3.5 Trends in DD for the two groups

**Figure 1:**
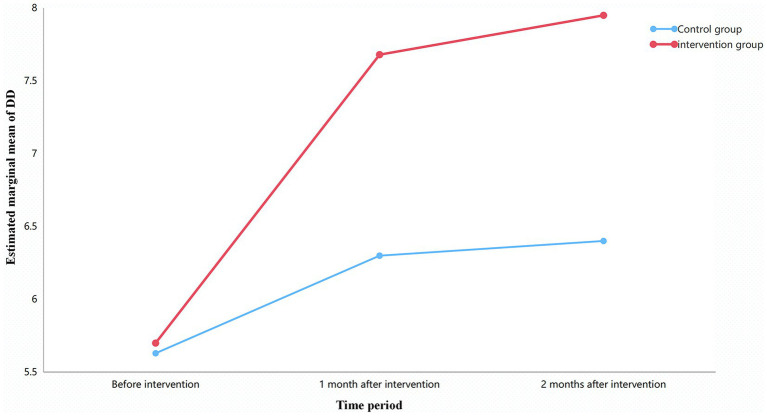
Trends in dietary diversity over time for both groups.

Repeated measures ANOVA revealed a significant interaction between group and time for Total DDS (*F* = 21.42, *p* < 0.001). The effect size was large (partial *η*^2^ = 0.215), indicating that the intervention group showed a substantially greater upward trend in dietary diversity over time compared to the control group (see Table 6 and Figure 1).

**Table 6:** Changes in dietary diversity over time for the two groups.

Grouping	Pre-intervention	After 1 month of intervention	After 2 months of intervention	*F* (group × time)	*p* (ANOVA)	Partial *η*^2^
Control group	5.63 ± 1.33	6.30 ± 1.14	6.40 ± 1.01	**—**	*—*	**—**
Intervention group	5.70 ± 1.24	7.68 ± 0.89	7.95 ± 0.78	**—**	**—**	**—**
*t*	−0.26	−6.03	−7.68	**—**	**—**	**—**
*p*	0.796	<**0.01**	<**0.01**	**21.42**	<**0.01**	0.215

Partial *η*^2^ = 0.215 indicates a large effect size. Bold values indicate statistically significant results at *p* < 0.05.

### 3.6 Trends in MNA-HF for two groups

**Figure 2:**
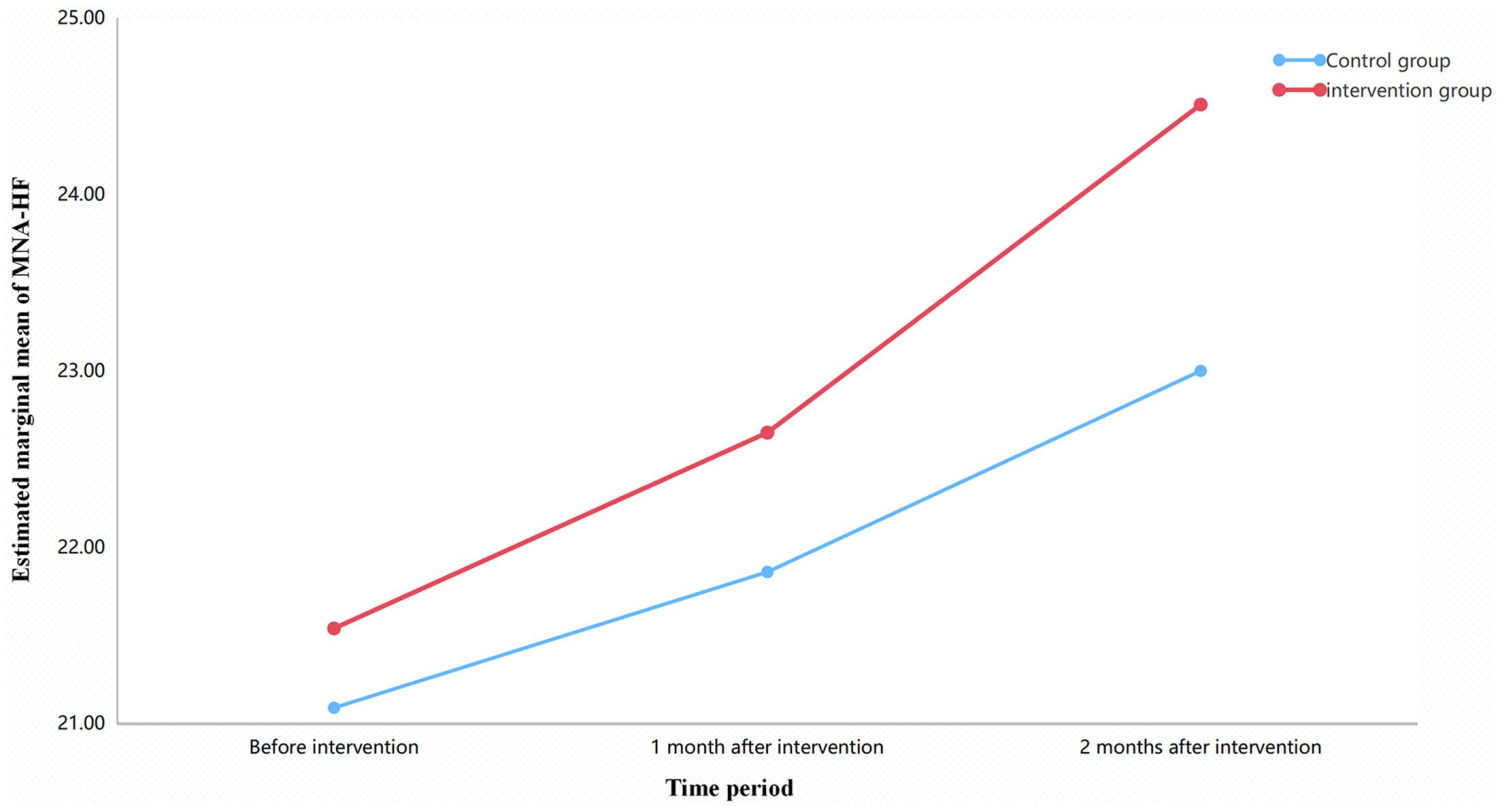
Trends in MNA-HF over time for both groups.

For MNA-HF scores, the interaction between group and time was not statistically significant (*F* = 1.66, *p* = 0.202), with a small effect size (partial *η*^2^ = 0.021). Both groups showed an increasing trend in scores over time, but the magnitude of change did not differ significantly between groups in the overall trend analysis, although the point-difference at 2 months was significant (as shown in Table 5) (see Table 7 and Figure 2).

**Table 7:** Changes in MNA-HF scores over time for two groups.

Grouping	Pre-intervention	After 1 month of intervention	After 2 months of intervention	*F* (group × time)	*p* (ANOVA)	Partial *η*^2^
Control group	21.09 ± 4.34	21.86 ± 3.81	23.00 ± 3.06	**—**	**—**	**—**
Intervention group	21.54 ± 3.67	22.65 ± 2.87	24.51 ± 2.27	**—**	**—**	**—**
*t*	−0.50	−1.04	−2.52	**—**	**—**	**—**
*p*	0.618	<**0.01**	<**0.014**	1.66	0.202	0.021

Partial *η*^2^ = 0.021 indicates a small effect size. Bold values indicate statistically significant results at *p* < 0.05.

### 3.7 Trends in BMI changes for two groups

**Figure 3:**
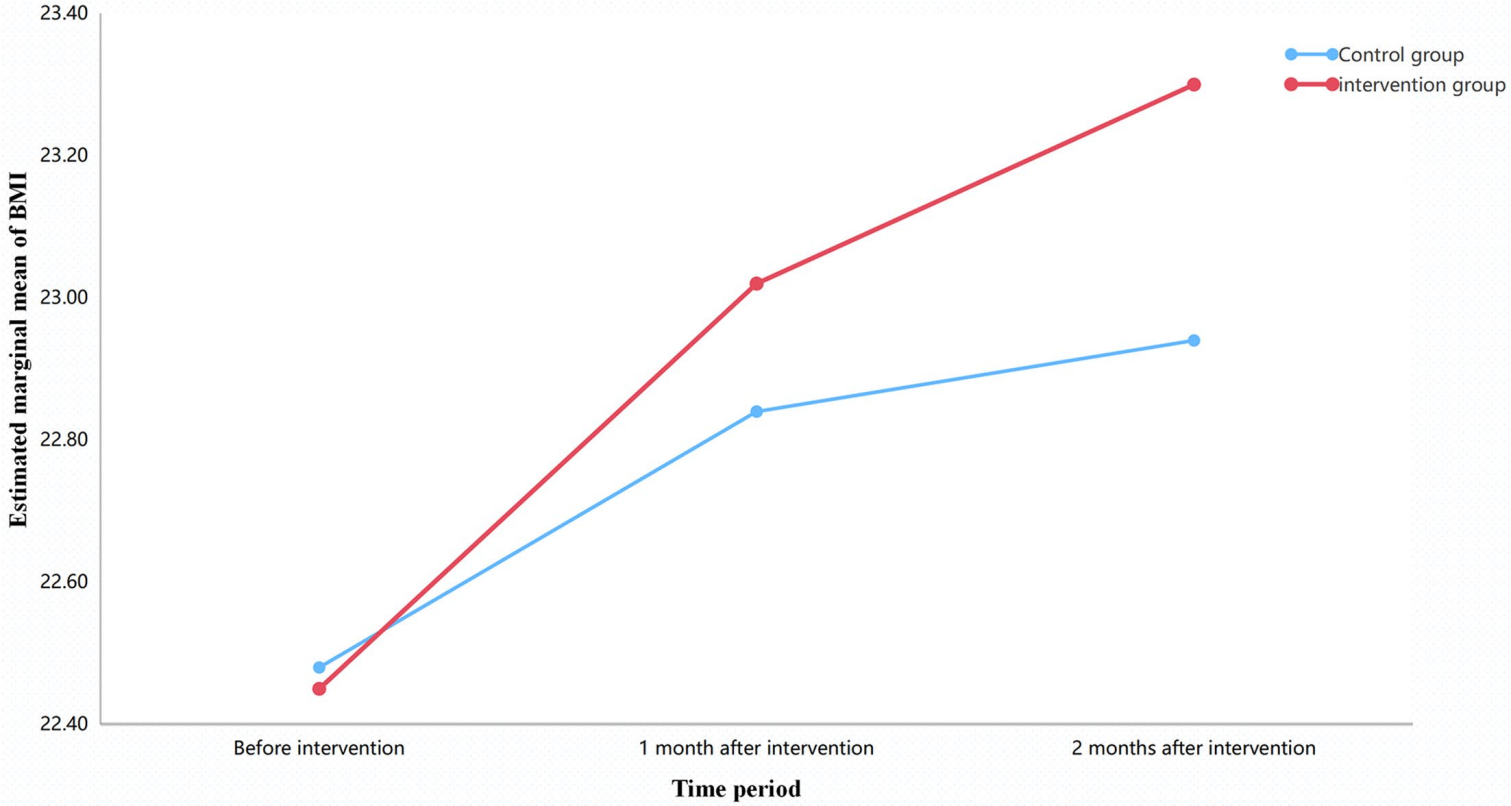
Trends in BMI over time for both groups.

The interaction between group and time for BMI was not statistically significant (*F* = 0.06, *p* = 0.813), and the effect size was negligible (partial *η*^2^ = 0.001). This indicates that the trajectory of BMI changes was similar between the two groups over the study period (see Table 8 and Figure 3).

**Table 8:** Changes in BMI over time for the two groups.

Grouping	Pre-intervention	After 1 month of intervention	After 2 months of intervention	*F* (group × time)	*p* (ANOVA)	Partial *η*^2^
Control group	22.48 ± 3.73	22.84 ± 3.45	22.94 ± 3.29	**—**	*—*	**—**
Intervention group	22.45 ± 3.43	23.02 ± 2.93	23.30 ± 2.80	**—**	**—**	**—**
*t*	0.03	−0.26	−0.52	**—**	**—**	**—**
*p*	0.978	0.795	0.604	0.06	0.813	0.001

Partial *η*^2^ = 0.215 indicates a large effect size.

### 3.8 Comparison of pre- and post-intervention BMI differences between the two groups

To further explore the impact on body weight, we analyzed the change in BMI relative to baseline. After 1 month, the increase in BMI did not differ significantly between groups (*p* = 0.097, *d* = 0.37). However, after 2 months, the intervention group showed a significantly greater increase in BMI compared to the control group (0.84 ± 0.82 vs. 0.47 ± 0.60 kg/m^2^; *t* = −2.35, *p* = 0.021). The effect size for this difference was medium (Cohen’s *d* = 0.52), suggesting a beneficial impact of the intervention on weight status over a longer duration (see Table 9).

**Table 9:** Comparison of pre- and post-intervention differences in BMI between the two groups.

Grouping	Control group	Intervention group	*t*	*p*-value	Cohen’s *d*	95% CI
Difference in BMI after 1 month	0.36 ± 0.47	0.57 ± 0.63	−1.68	0.097	0.37	0.07 ~ 0.81
Difference in BMI after 2 months	0.47 ± 0.60	0.84 ± 0.82	−2.35	**0.021**	0.52	0.05 ~ 0.98

Cohen’s *d* indicates effect size (0.2 = small, 0.5 = medium, 0.8 = large). CI, confidence interval. Bold values indicate statistically significant results at *p* < 0.05.

## 4 Discussion

This study provides novel evidence that a DDS-guided, tiered nutrition education program can effectively enhance dietary diversity and nutritional status among elderly patients with chronic heart failure (CHF). By translating dietary variety into measurable targets and offering individualized reinforcement, the intervention advances traditional dietary counseling from a one-size-fits-all approach to a precision-oriented model that is both practical and scalable in clinical settings. The findings highlight the potential of structured dietary diversification as a feasible strategy for improving nutritional quality and supporting self-management in elderly CHF populations, particularly in resource-limited contexts.

### 4.1 Effectiveness of nutrition education on dietary diversity

Nutrition education is the most basic, cost-effective, and efficient way to address public nutrition and health issues (44). It is also the practical application of health promotion theories, tactics, and approaches in the field of public nutrition. Studies show that nutrition education-based nursing interventions can lower readmission rates and ease the cost burden of heart failure patients, saving around $3.9 million over 4 years (45). Crucially, our study confirms that among elderly CHF patients who consume orally, DD nutrition education improves dietary diversity. Our statistical analysis revealed a large effect size for the improvement in Total DDS over time (partial *η*^2^ = 0.215), and a very large effect size for the difference between groups at 2 months (Cohen’s *d* = 1.72). This indicates that the intervention produced a substantial and robust change in patients’ dietary behaviors. This result is consistent with a study on nutrition and health education intervention among pregnant women in Malawi’s rural areas (46). At the same time, relevant studies show that nutrition education can raise the levels of dietary diversity in groups like kids and teenagers (47, 48). Additionally, DD nutrition education raises patients’ health consciousness and promotes better dietary and lifestyle choices (49), therefore enhancing life quality (15). DD nutrition education thus provides older patients with easy-to-understand and highly actionable nutritional advice, even in the absence of access to professional nutritionists.

### 4.2 Improvement in nutritional status (MNA-HF)

The intervention group demonstrated a significant improvement in MNA-HF scores compared with the control group, indicating enhanced overall nutritional status after 2 months of dietary diversification education. The calculated effect size for this change was medium (*d* = 0.56), reflecting a practically meaningful improvement rather than a small statistical fluctuation. Clinically, poorer nutritional status has been consistently associated with worse outcomes in patients with heart failure, including higher all-cause mortality and adverse clinical events. A systematic review and meta-analysis reported that poor nutritional status assessed by multidimensional screening and assessment tools—including the Mini Nutritional Assessment (MNA) and related indices—predicted all-cause mortality in patients with heart failure (50). In addition, malnutrition determined by established criteria such as the GLIM framework has been associated with an increased risk of all-cause death or heart failure-related readmission after discharge (51). These findings suggest that even modest improvements in nutritional status, as reflected by MNA-HF scores, may translate into meaningful clinical benefits, underscoring the relevance of the observed effect in this study.

However, 1 month after the intervention, dietary diversity levels significantly improved (large effect size), but this did not result in immediate statistically significant improved nutritional status. This disparity might result from older CHF patients receiving oral feedings experiencing a physiological loss in organ function, which lowers intake. Therefore, short-term changes in nutritional status might not be immediately noticeable, even with improved DD levels. This implies that patients may benefit more from a longer intervention. Additionally, dietary advice should be given often during the duration of treatment. According to research, a number of problems could occur during the nutritional management process, making it more difficult for patients to maintain a healthy nutritional status (52). As a result, it is crucial to identify these obstacles as soon as possible throughout the intervention.

### 4.3 Impact on body mass index (BMI)

The BMI of patients can also be improved via DD intervention. The trend chart illustrates how patients’ BMI first rises quickly before slowing down, which is in line with Jin’s (41) study. Although the overall trend analysis did not show a significant interaction, the pairwise comparison at 2 months revealed a significantly greater increase in BMI in the intervention group compared to controls, with a medium effect size (Cohen’s *d* = 0.52). A BMI of 25.0 to 29.9 kg/m^2^ has been found to be an independent protective factor against adverse cardiovascular events in CHF patients throughout follow-up (*p* < 0.05). Patients with normal or low body weight have a worse prognosis and are more likely to experience unfavorable cardiovascular events than those who are overweight. The obesity paradox seen in HF patients (53) may be the cause of this result. Higher BMI indicates greater metabolic reserve, lower sympathetic activity, decreased responsiveness to neuroendocrine stimuli, and partial mitigation of HF’s catabolic effects, which may provide metabolic buffering against disease progression (54). These benefits of the obesity paradox in HF patients may result from these factors. Improving dietary density (DD) may raise BMI in this study sample because of their generally low BMI.

Although the observed increase in BMI among participants in the intervention group suggests improved nutritional status, this indicator must be interpreted cautiously in patients with chronic heart failure. BMI can be affected by fluid retention and peripheral edema, which are common in CHF and may artificially elevate body weight independent of actual nutritional gain. In the present study, all participants were clinically stable at baseline and received standardized diuretic management throughout the intervention period, which helps reduce the confounding influence of fluid status. Nevertheless, the potential impact of subclinical fluid shifts cannot be completely excluded. Future studies should incorporate more sensitive measures of body composition, such as bioelectrical impedance analysis or dual-energy X-ray absorptiometry (DXA), to better distinguish between true nutritional improvement and fluid-related weight change.

### 4.4 Potential mechanisms

By boosting appetite, increasing food intake, and encouraging increased consumption of macronutrients and micronutrients, DD intervention may enhance the nutritional status of elderly CHF patients receiving oral feeding (29, 55, 56). Furthermore, Huang et al.’s (57) research suggests that elevated DD levels enhance the variety of gastrointestinal probiotics, which is linked to patients’ improved gastrointestinal function and, in turn, improved nutrient absorption. Additionally, pertinent research suggests that by increasing vascular endothelial growth factor (58). Lowering risk factors (such as diabetes, hypertension, etc.) (59–61), and lowering coronary atherosclerosis (62), DD may improve prognosis and delay the progression of the disease in older CHF patients.

## 5 Limitations

The following limitations should be acknowledged. First, although the clinical environment and care pathways remained stable throughout the study period, the non-randomized, non-concurrent control design may still introduce residual temporal bias. This approach was adopted to avoid cross-contamination within shared ward settings, and consistency in staff, protocols, and ward routines was maintained; nevertheless, unmeasured time-varying factors cannot be fully excluded. Second, despite comparable baseline characteristics with negligible effect sizes, the absence of randomization may allow potential selection bias. Participants were enrolled consecutively, which could lead to unmeasured differences such as motivation or family support. While causal inference should be interpreted cautiously, the consistent improvements in DDS and MNA-HF provide preliminary evidence of practical effectiveness that merits validation in larger studies. Third, the use of a qualitative Food Frequency Questionnaire (FFQ) assessed food variety but not quantitative nutrient intake. This simplified method was chosen for feasibility in elderly CHF patients, though it limits evaluation of exact nutrient adequacy. Fourth, the two-month follow-up period was relatively short, preventing assessment of long-term sustainability and clinical outcomes. Future randomized or longitudinal studies with extended follow-up are warranted to confirm the durability and prognostic relevance of dietary diversification interventions. Finally, this was a single-center study conducted in Guizhou province, where distinct dietary habits and socioeconomic conditions may limit generalizability to other populations and healthcare settings; therefore, multicenter trials across diverse regions are needed to verify external applicability.

## 6 Conclusion

This study shows that dietary diversity interventions can effectively increase dietary diversity levels and improve nutritional status in older patients with chronic heart failure (CHF) who can eat orally (as evidenced by more favorable BMI differences and increased MNA-HF scores). Additionally, the format and substance of this intervention demonstrate good clinical application and feasibility, and it is suited to the peculiarities of senior patients, making it worthy of clinical promotion.

## Data Availability

The datasets presented in this study are not readily available because of privacy and ethical restrictions involving human participants. Requests to access the datasets should be directed to the corresponding author.
